# Development and testing of a transcatheter heart valve with reduced calcification potential

**DOI:** 10.3389/fcvm.2023.1270496

**Published:** 2023-12-06

**Authors:** Hellmuth Weich, Lezelle Botes, Anton Doubell, Johan Jordaan, Angelique Lewies, Prennie Marimuthu, Johannes van den Heever, Francis Smit

**Affiliations:** ^1^ Division of Cardiology, Department of Medicine, Faculty of Medicine and Health Sciences, Stellenbosch University, Cape Town, South Africa; ^2^ Department of Health Sciences, Central University of Technology, Bloemfontein, South Africa; ^3^ Department of Cardiothoracic Surgery, Robert W.M. Frater Cardiovascular Research Centre, University of the Free State, Bloemfontein, South Africa

**Keywords:** TAVI, transcatheter heart valve, decellularized pericardium, low-dose glutaraldehyde, calcification, ovine model

## Abstract

**Introduction:**

Patients from developing countries who require heart valve surgery are younger and have less access to open heart surgery than those from developed countries. Transcatheter heart valves (THVs) may be an alternative but are currently unsuitable for young patients because of their inadequate durability. We developed and tested a THV utilizing two new types of decellularized bovine pericardial leaflets in an ovine model.

**Methods:**

The two decellularized tissues [one with a very low dose (0.05%) of monomeric glutaraldehyde (GA) fixation and detoxification (DF) and the other without glutaraldehyde (DE)] were compared to an industry standard [Glycar—fixed with the standard dose (0.625%) of glutaraldehyde]. THVs were manufactured with the three tissue types and implanted in the pulmonary position of nine juvenile sheep for 180 days. Baseline and post-explantation evaluations were performed to determine the hemodynamic performance of the valves and their dynamic strength, structure, biological interaction, and calcification.

**Results:**

Heart failure occurred in one animal due to incompetence of its Glycar valve, and the animal was euthanized at 158 days. The gradients over the Glycar valves were higher at the explant than at the implant, but the DE and DF valves maintained normal hemodynamic performance throughout the study. The DF and DE tissues performed well during the mechanical testing of explanted leaflets. Glycar tissue developed thick pannus and calcification. Compared to Glycar, the DF tissue exhibited reduced pannus overgrowth and calcification and the DE tissue exhibited no pannus formation and calcification. All tissues were endothelialized adequately. There was a striking absence of host ingrowth in the DE tissue leaflets, yet these leaflets maintained integrity and mechanical function.

**Conclusion:**

In the juvenile sheep THV model, Glycar tissue developed significant pannus, calcification, and hemodynamic deterioration. Using a very low dose of monomeric GA to fix the decellularized bovine pericardium yielded less pannus formation, less calcification, and better hemodynamic function. We postulate that the limited pannus formation in the DF group results from GA. Bovine pericardium decellularized with our proprietary method resulted in inert tissue, which is a unique finding. These results justify further development and evaluation of the two decellularized tissue types in THVs for use in younger patients.

## Introduction

Transcatheter aortic valve implants (TAVIs) have revolutionized the management of elderly patients with severe aortic stenosis (AS) and have now surpassed surgical aortic valve replacement (SAVR) as the preferred treatment modality in First World countries (1). They provide a less invasive and more durable alternative for patients who may well otherwise have struggled to cope with the trauma of SAVR. TAVI, as an alternative procedure to SAVR, has proven to be safe and efficient for the whole risk spectrum of patients, including patients deemed to be inoperable (2), high risk (3, 4), intermediate risk (5, 6), or even low risk (7, 8). All TAVIs and other transcatheter heart valves (THVs) are biological valves of either porcine or bovine origin. These materials have been shown to deteriorate and calcify in younger patients in large surgical series (9–11). Hence, a crucial caveat in all these randomized TAVI trials was that the participants were elderly, with mean age ranging from 74 to 83 years. In view of this, the European Society of Cardiology (ESC) guidelines recommend that TAVIs be reserved for patients ≥75 years or with high surgical risk and mechanical valves should be used in patients younger than 60 years when SAVR is performed (12). In the United States, only 18% of SAVR procedures and 30% of mitral valve replacements utilize mechanical valves, as the mean age of surgical valve recipients is 67 years (13, 14). This is in stark contrast to studies from Africa, where the average age of heart valve surgery candidates was 19 years in Ethiopia (15) and Uganda (16), 26 years in the Côte d'Ivoire (17), 27 years in Nigeria (18), and 42 years in South Africa (19). In these age groups, the recommendation is that all patients receive mechanical prostheses, but the problems associated with lifelong anticoagulation treatment are well documented (20–22). If one adds to this the fact that rheumatic heart disease (RHD) remains the most prevalent cause of significant heart valve disease in the world (23) and that there is a huge unmet need for surgery for these patients (24), there is a lot of potential for improved THVs to address these needs (23, 24). In this context, a durable bioprosthetic transcatheter valve with reliable and reproducible deployment is an attractive future alternative. Although transcatheter valve implantation in younger patients faces design, deployment, and structural challenges, the durability of bioprosthetic or tissue-engineered leaflet alternatives remains the Achilles’ heel of this concept. The durability of bioprosthetic valves in patients under 40 years is poor (25–27) and therefore makes this type of valve inappropriate for the largest group of potential recipients of heart valves in the world (23). Improved durability of bioprosthetic material is therefore imperative. Since the very first valve replacements (28, 29), the only landmark improvement in bioprosthetic tissue fixation has been the switch from formaldehyde to glutaraldehyde (GA) (30). However, GA fixation has been plagued with valve deterioration and calcification, both as a result of ongoing immune processes and toxicity associated with the aldehydes. Various anti-calcification techniques have been investigated (31–33), but an ideal process for producing durable biological valves remains elusive. The process of biological valve deterioration is complex and includes remnant tissue immunogenicity, inflammatory cell infiltration, toxicity of GA fixation, mechanical damage, calcification, lack of repair, and pannus overgrowth (34). Therefore, the development of tissue-engineered alternatives and biological or artificial scaffolds has become a major research focus (35).

The Robert W M Frater Cardiovascular Research Centre (Frater Centre) at the University of the Free State (UFS) has been involved in the development of a biological tissue scaffold with reduced immune response from the host while maintaining sufficient dynamic strength to survive in the harsh mechanical stress environment to which heart valves are exposed. These processes can be broadly divided into decellularization, fixation, and detoxification. After evaluating the contributions of various decellularization steps and combinations, we have developed a unique method to decellularize bovine pericardium (BP) without significantly altering its strength and collagen structure (36, 37). This decellularized (DE) tissue performed well in 6-month implants in ovine aortas and pulmonary arteries, with less pannus formation, limited calcification, and adequate strength (38). Despite these results, it is not known whether the DE tissue would be strong enough to function in a high mechanical stress environment such as a THV. Therefore, additional cross-linking with GA may be required, and we have developed an additional decellularized, fixed, and detoxified (DF) tissue. The hypothesis is that GA has detrimental effects on tissue and should be limited as much as possible. This tissue was therefore fixed with a very low dose of GA, which was combined with a proprietary amino acid detoxification process, which in turn indicated no toxicity, excellent cross-linking, resistance to calcification, and porosity, allowing host cell recellularization in a subcutaneous rat model (39). To improve cross-linking, we utilized monomeric GA, which has been shown to produce less calcification (40).

We have developed a balloon expandable THV designed to be manufactured with bovine pericardial leaflets (41–45). This THV provided the platform on which we tested the different pericardial leaflets.

This study aimed to test DE, DF, and an industry standard bovine pericardium (Glycar, Glycar Pty Ltd, Irene, South Africa) in THVs implanted in the right ventricular outflow tract (RVOT) of juvenile sheep for 180 days. We compared the hemodynamic performance, structural degeneration and tissue strength, host tissue repopulation, inflammatory response, and calcification.

## Materials and methods

### Study design

**Figure 1:**
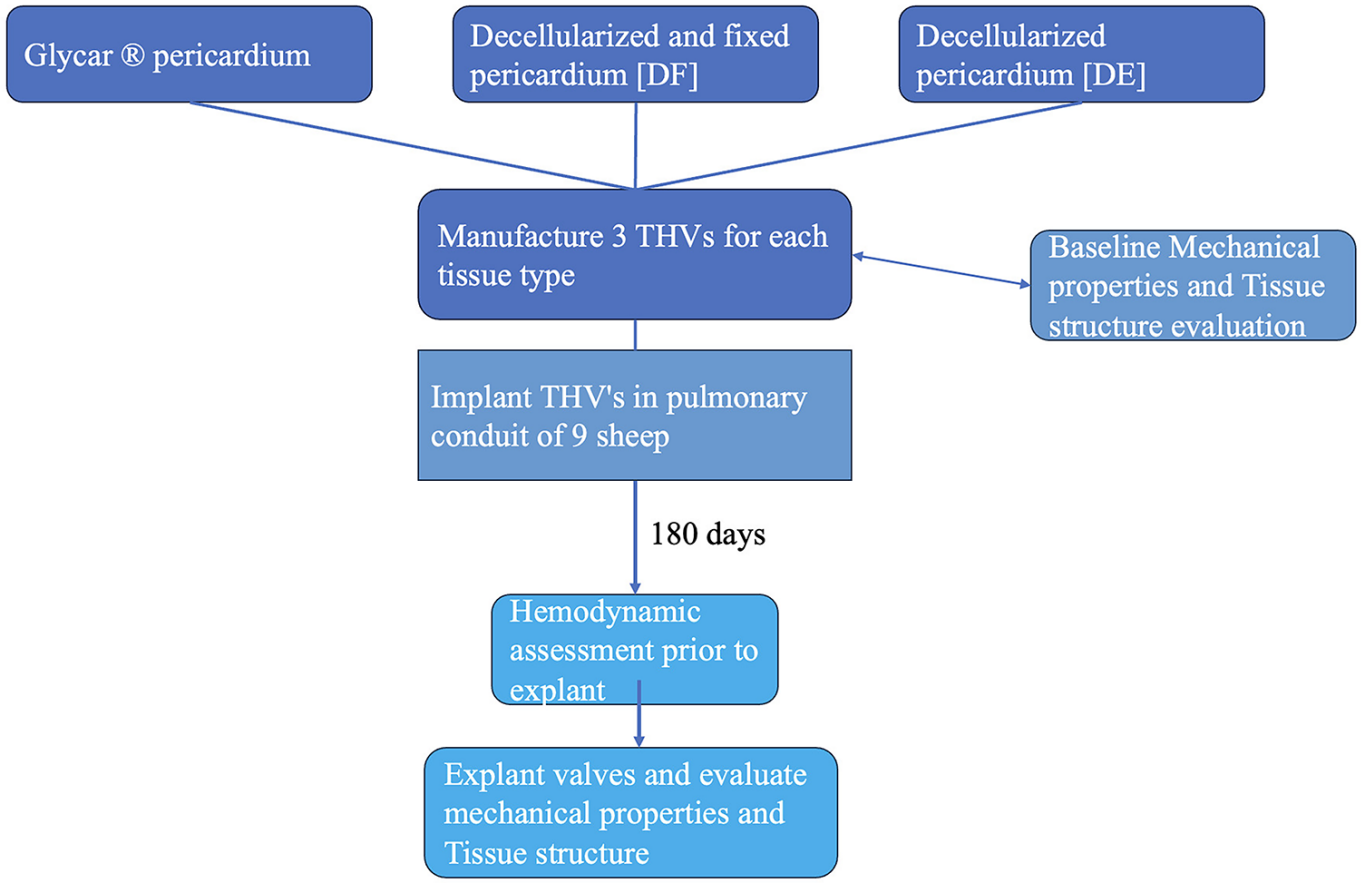
Schematic representation of the study design.

The study was conducted as a prospective analytical cohort experimental study. Baseline and post-explantation tissue data were documented and compared between groups. Three THVs per group were constructed from bovine pericardium: either decellularized (DE) according to our proprietary method (*n *= 3) (37, 38) or decellularized, fixed, and detoxified (DF) (*n *= 3) or an industry standard (Glycar) (*n *= 3). The valves were then implanted as interposition grafts in the main pulmonary artery (MPA) of juvenile Merino sheep (*n *= 3 per group) for 180 days. Echocardiography was performed at implantation and after 3 and 6 months to monitor valve function. Valve leaflet tissue at the explant was evaluated macroscopically and radiographically. Dynamic strength testing was performed using tensile strength (TS) and flexibility [Young's modulus (YM)] analyses, and morphological evaluation included hematoxylin and eosin (H&E) staining, von Kossa staining, Verhoeff–Van Gieson elastic staining (EVG), scanning electron microscopy (SEM), and transmission electron microscopy (TEM). A schematic representation of the study design is presented in Figure 1.

The Animal Ethics Committees of the University of the Free State (UFS-AED2016/0008/2020/21) and the University of Stellenbosch (SU-ACUD15-00120) approved the project.

### Experimental animals

The juvenile ovine model was selected for testing the valve *in vivo* because of the similarity of the animal's anatomy and hemodynamics to humans (46, 47) and because the model is approved by both the FDA and CE Mark (48). The exact definition of what constitutes a juvenile sheep is lacking, and in line with others (49–51), we elected to use animals that were 6 months of age. This negates the excessive growth of younger animals while still providing an accelerated calcification model for the valves. Furthermore, they are considered the ideal valve calcification model (52). Nine juvenile Merino sheep of 6 months of age and weighing between 40 and 45 kg were used to achieve a satisfactory match between valve size and pulmonary artery size. All animals were vaccinated, treated against ecto- and endoparasites, and subjected to a complete blood count prior acceptance for surgery. All animal experiments and surgical procedures were performed in compliance with the Guide for the Care and Use of Laboratory Animals published by the US National Institutes of Health (53) and guided by the South African National Standard 10386:2021 for the care and use of animals for scientific purposes.

### Tissue processing

Three groups of bovine pericardial leaflet constructs were used in the study:

(i) Glycar bovine pericardial tissue: Using EnCap technology, Glycar bovine pericardial tissue was selected as the industry standard since it has an established track record in the field (54, 55). Furthermore, we have used Glycar as a comparator in our previous work (37, 38) and deemed it the most suitable tissue available for use as a control. This tissue was GA-tanned (0.625%), formaldehyde (4%)-sterilized, detoxified with propylene glycol (100%), and stored in propylene oxide (2%).
(ii) Decellularized (DE) tissue: A decellularized bovine pericardial scaffold was prepared by decellularizing fresh bovine pericardium using a proprietary process developed by our group. This process combines the use of osmotic shock, a multi-detergent solution, delipidation with ethanol, and sterilization in an antibiotic and anti-mycotic solution according to a patented method (Patent: 16702008.0-1455, EU, 2017) (38).
(iii) Decellularized-fixed (DF) tissue: Bovine pericardium decellularized as in (ii), then fixed with 0.05% monomeric GA (Polysciences, Inc., USA), and detoxified with an amino acid solution containing 0.1 M glycine (39).

Decellularization was evaluated by measuring the DNA content and inspecting H&E staining to confirm acellularity. DNA was extracted from the tissue using a Quick-DNATM Miniprep Plus Kit (Zymo Research, USA), and the complete decellularization of the tissue was confirmed by measuring the DNA content (ng DNA/mg tissue) using a BioDrop spectrophotometer (Biochrom Ltd., Cambridge, UK). After processing, the tissue was confirmed to be culture-negative (anaerobic and aerobic bacteria, fungi, and yeast) by a registered pathology laboratory (PathCare Veterinary Laboratory Bloemfontein, South Africa).

### Valve construction

**Figure 2:**
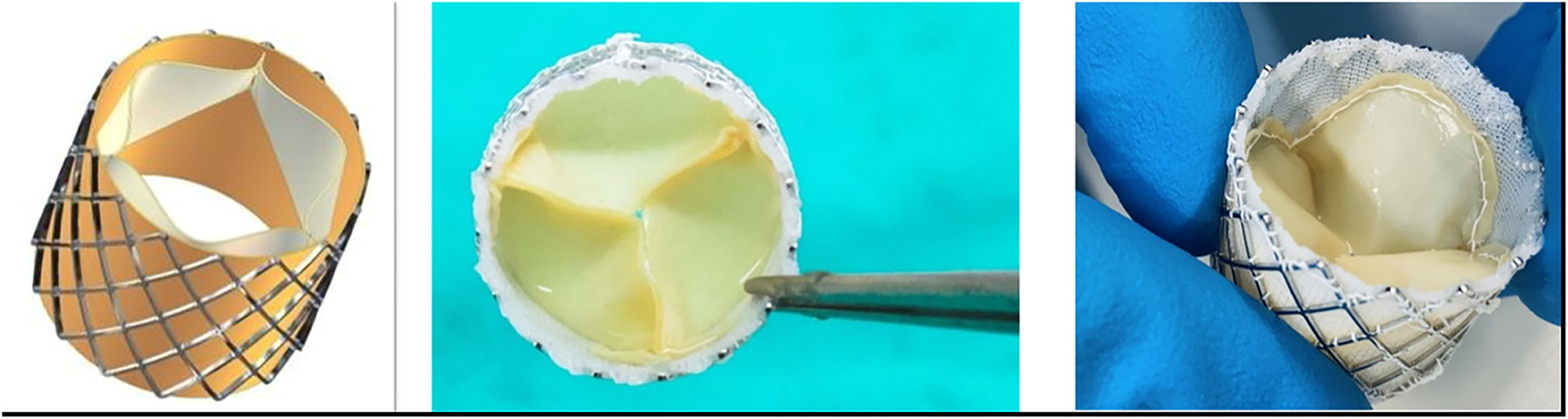
THV. On the left is a computer design of the valve that was developed using finite-element analysis (41–44). Note the balloon expandable stent frame with pericardial leaflets and a braided PTFE hemostatic cuff on the inside of the stent. On the right are two photographs of a manufactured valve.

A stainless-steel stent platform for the THV prosthesis was developed and tested *in vitro* and virtually to optimize the design and geometry of the leaflets (41–45). The valves were constructed in an ISO 14385-compliant clean room (Next Biosciences, Johannesburg, South Africa). A hemostatic cuff made from braided PTFE (Bard, Tempe, AZ, USA) was sutured onto the inside of the stent with individual self-locking sutures using CV-7 monofilament Gore-Tex (W.L. Gore & Associates, DE, USA). Three sets of valves were prepared for comparison using leaflet tissue supplied by Glycar and DE and DF pericardium scaffolds supplied by the Frater Centre. A leaflet template was manufactured to cut three identical-sized leaflets from a single sheet of pericardium, and the leaflets were then sutured with CV-7 Gore-Tex sutures onto the stent frame. A 22-mm THV valve was constructed according to the design parameters developed by our group (Figure 2).

Swabs for microbiology testing were taken from the covered stent and the completed valve before final storage in 2% propylene oxide (Glycar leaflets) or an 85% glycerol/15% ethanol solution (decellularized leaflets), as well as a tissue sample from the pericardial tissue used for the construction of the valve leaflets.

### Surgical implantation of transcatheter heart valves

The THV prosthesis was aseptically removed from the storage solution in the theater and rinsed in sterile 0.9% saline solution for 3–5 min. To expose the tissue to the mechanical forces of a transcatheter implant, it was crimped onto a 23-mm Z-Med balloon (Numed, NY, USA) using a commercially available crimping tool. The crimped diameter was confirmed to be small enough to fit through an 18-French sheath by inserting it into a dedicated Perspex tube with a 6 mm inner diameter. The crimped valve was then fully deployed with balloon inflation within a 22-mm Jotec FlowWeave Bioseal woven polyester vascular graft (JOTEC GmbH, Germany), secured with three polypropylene sutures at each end to prevent migration. The vascular graft was 3 mm longer than the THV on either side (total length ± 24 mm) to enable suturing and was then implanted as an RVOT conduit.

Recipient sheep were sedated (morphine sulfate 1 mg/ml, 0.2–0.5 mg/kg IM; ketamine hydrochloride 100 mg/ml, 5–15 mg/kg IM; Robinul 4 mg/2 ml, 0.005–0.01 mg/kg IM), anesthetized (Propofol 10 mg/ml, 2–6 mg/kg IV, Esmeron 50 mg/5 ml, 0.5 mg/kg IV), intubated, and ventilated (Isofor 2.5%, 1%–3% inhalation). A central venous line (CVP) was inserted in the left jugular vein, an arterial line was placed in the left carotid artery for hemodynamic monitoring, and the left carotid artery was cannulated for bypass. A left thoracotomy was performed, and the fourth rib was removed. Removal of the rib allows for a wider field of view during surgery and better echo windows at the follow-up. The right atrium was cannulated to achieve cardiopulmonary bypass (CPB), allowing the surgical procedure to be performed on a beating heart. The main pulmonary artery was clamped and transected, native pulmonary valve leaflets were removed, and the THV conduit was implanted as an interposition graft using continuous 4/0 prolene sutures in the pulmonary artery. The animal was weaned off CPB, hemostasis was secured, and an intrathoracic sonar was performed to confirm adequate valve functioning. The pressure gradient across the conduit was measured through needle insertion on either side of the conduit. A single chest drain was inserted on the left side, and all incisions were closed. The animal was extubated when awake and breathing on its own, and the chest drain and monitoring lines were removed soon after. The sheep were moved back to the indoor holding pens with companion sheep in adjacent pens. Precisely, 5 ml of Depomycin (MSD Intervet) as a broad-spectrum antibiotic and 0.1–0.2 mg/kg morphine sulfate (Fresenius Medical Care, South Africa) as pain medication were administered intramuscularly twice daily for 5 days after surgery, and the animals were followed up and monitored daily for 6 months until they were killed; the valves were explanted for further analysis of the leaflet tissue. A piece of the adjacent pulmonary artery was excised and sent for microscopy and culture to exclude infection (negative in all cases).

### Clinical performance of implanted valves

Echocardiography was performed just after implanting the conduit, at days 30 and 90 post-surgery, and just before killing them using a GE Vivid-Q portable sonar machine. Over the study period, implanted valves were evaluated for transvalvular gradients, valve regurgitation, calcification, and possible aneurysm formation.

At the end of the study period, the animals were again anesthetized as described above and analgesia was administered (morphine sulfate 0.1–0.2 mg/kg); the chest was opened, hemodynamic pressures were measured over the valve by needle insertion, and intrathoracic echocardiography was performed. Animals were then killed by injecting a bolus of 10 ml of 15% hyperosmolar potassium chloride directly into the right atrium to cause cardiac arrest and then exsanguinated, and the valves were removed for further analyses.

### Tissue analysis

Mechanical properties, tissue histology, and ultrastructure of a representative piece of pericardium used to manufacture each valve were analyzed (baseline analysis) and compared to results of similar analyses on the tissue leaflets after 180 days in the sheep (explant analysis).

#### Mechanical properties

Pericardial thickness was measured prior to surgical implantation and repeated after explantation using an ElectroPhysik MiniTest thickness gauge (Cologne, Germany). Samples were cut into 5-mm-wide strips, three to five thickness measurements were taken over the central section of the test strip, and then the average was calculated.

The dynamic strength test (tensile strength and Young’s modulus analyses) of the bovine pericardium tissue was uniaxially performed at room temperature by an automated and computerized TS testing apparatus (Lloyds LS100 Plus, IMP, South Africa). Preimplantation tissue (5 cm × 5 mm) and tissue at the explant (5 mm wide) were cut. The average thickness was then measured, and the tissue sample was gradually stretched between two grips until the breaking point was achieved. Force was calculated using a 500-N load cell. The tensile strength (MPa) and Young's modulus (MPa) were calculated from the stress–strain curve using Nexygen Plus 3 software (Lloyd Instruments, IMP, Johannesburg, South Africa).

#### Histology

A tissue sample from each pericardial patch used for leaflet construction was collected in 4% buffered formalin, dehydrated in graded alcohol steps, cleared in xylene, embedded in a paraffin wax block, sectioned, and stained according to standard protocols for H&E, von Kossa, and Verhoeff–Van Gieson staining for histological evaluation (56). The H&E-stained samples were evaluated for pannus formation, which was classified into three categories: none, non-confluent, and confluent pannus (covering the whole segment evaluated and both sides of the leaflet).

Pericardial tissue samples for electron microscopy evaluation were collected in 3% buffered GA and processed according to standard protocols for SEM and TEM evaluations (57).

### Statistical analysis

Due to the limited sample size, non-parametric analysis was used. Statistical analyses were performed using GraphPad Prism version 9.3.1. Continuous values were subjected to a Kruskal–Wallis (KW) multiple comparison analysis with Dunn's *post-hoc test* to compare individual groups. For two group comparisons, the Mann–Whitney *U*-test was used to measure differences between independent groups, and the Wilcoxon singed-rank test was used to measure differences between dependent groups.

## Results

**Figure 3:**
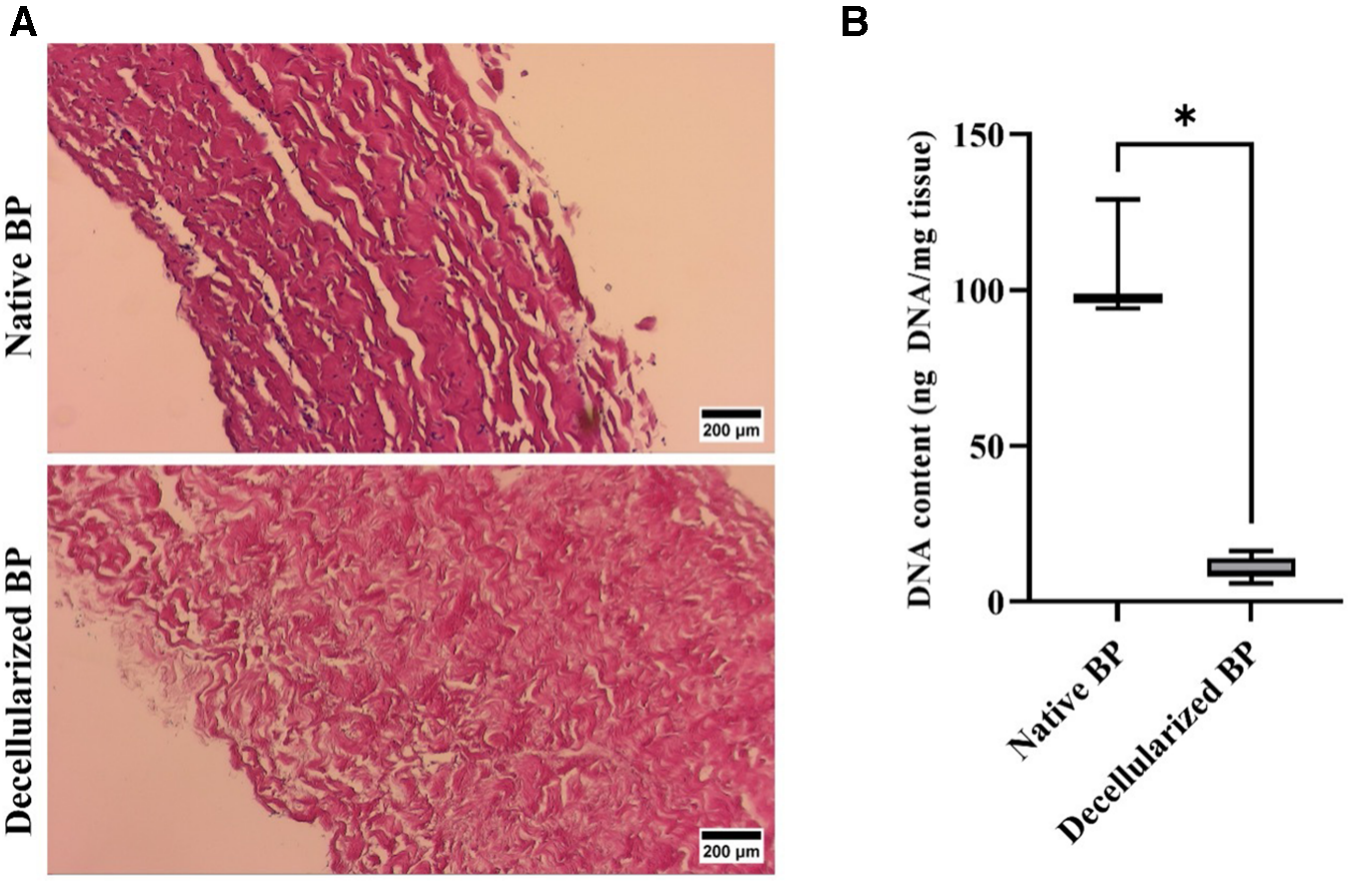
Confirmation of decellularization of bovine pericardium. (**A**) Representative light microscope images for H&E-stained tissue, indicating the presence of cells in the native BP and the complete removal of cells following decellularization (magnification 100×, scale 200 µM). (**B**) DNA content of the native and decellularized bovine pericardium (*n *= 6). * *p* < 0.05. The DNA content was well below the required 50 ng/mg tissue (71).

Adequate decellularization was confirmed for both DE and DF tissues prior to implantation (Figure 3).

**Figure 4:**
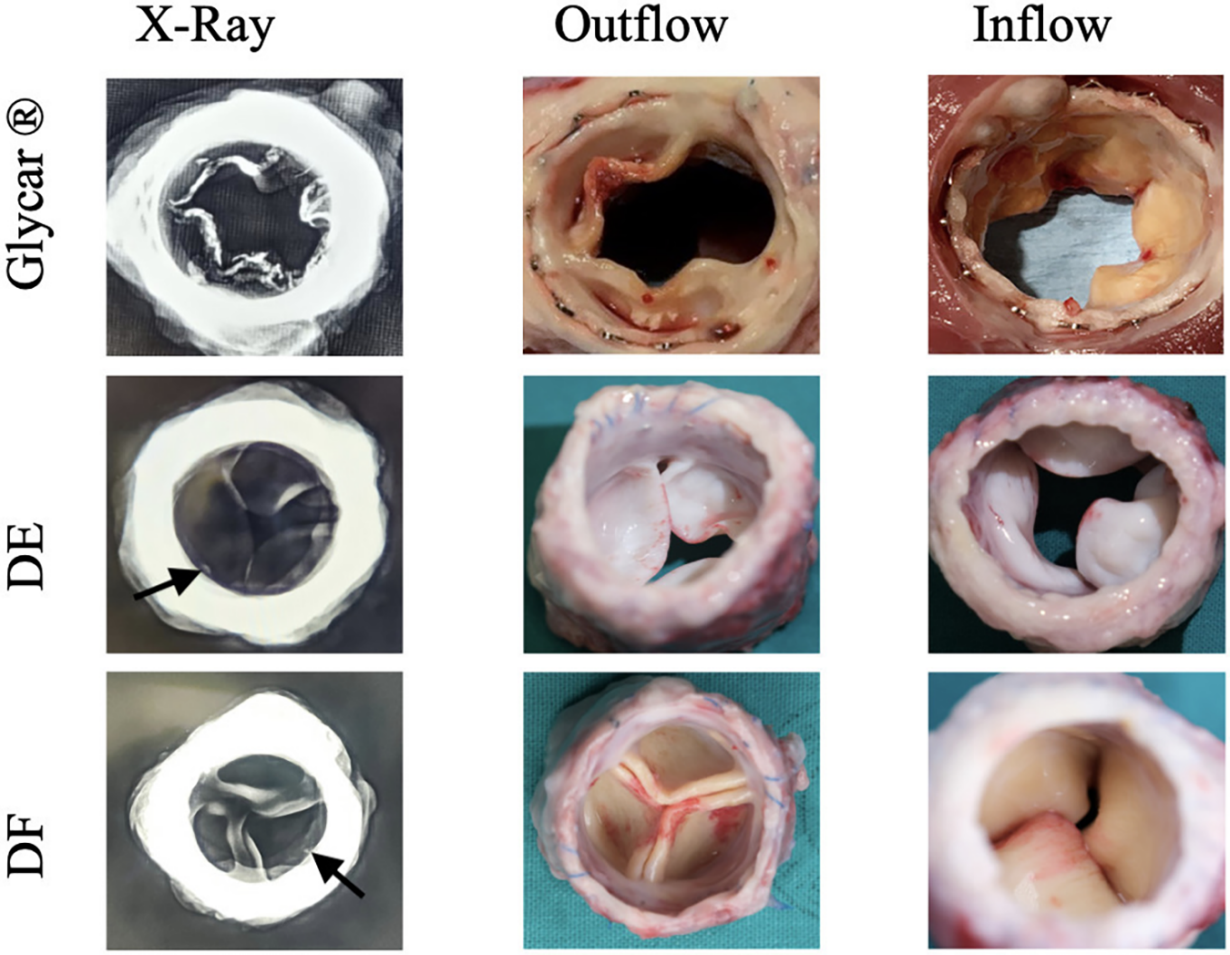
Representative X-ray images and photographs of the three differently processed valves at explantation. Note the calcification visible on the X-ray in the Glycar valve. The outflow view of this valve shows extensive pannus overgrowth, which has fixed one leaflet in the open position. Calcified nodules are visible on the inferior leaflet in the image. The decellularized (DE) and decellularized, low-dose glutaraldehyde-fixed, and detoxified (DF) bovine pericardium leaflets show no overt calcification, although there are possibly punctuated calcium spicules near the sewing margin (arrows), which may represent suture trauma. Glycar is the commercially available Glycar bovine pericardium fixed with conventional high-dose GA.

**Figure 5:**
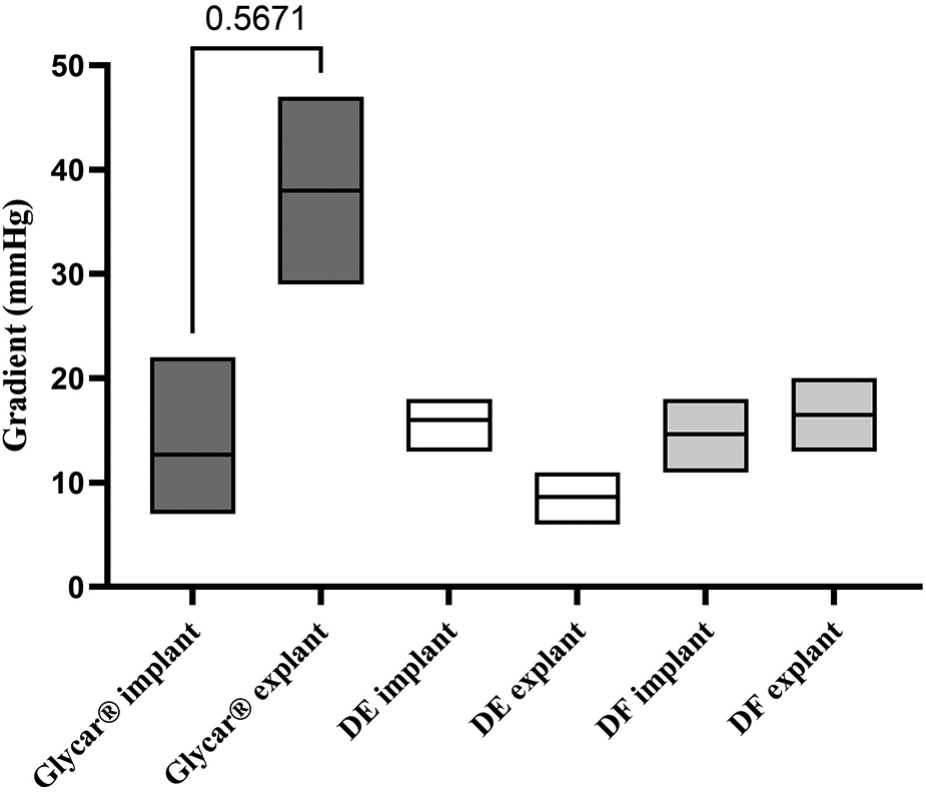
Invasively measured gradients (means and ranges) across the valves at the time of implantation compared to just prior to explantation. Note the unchanged gradients in DF and DE tissue valves. The change in gradients over the Glycar tissue valves was not statistically significant (*p *= 0.5671). DE denotes decellularized bovine pericardium; DF denotes decellularized, low-dose glutaraldehyde-fixed, and detoxified bovine pericardium; Glycar is the commercially available Glycar bovine pericardium fixed with conventional high-dose GA.

Valves were successfully implanted into 10 animals. One animal developed acute respiratory decompensation immediately post-operatively and was sacrificed. The other nine animals (three per tissue type) survived the procedure. All animals survived for 180 days except for one sheep with the Glycar valve, which developed heart failure and was euthanized at 158 days. The heart failure was due to a calcified prosthetic pulmonary valve with one of its leaflets overgrown by pannus and fixed in the open position (Figure 4). Endocarditis was excluded with blood cultures, c-reactive protein (CRP), and histology of this valve. The other valves were found to function adequately and competently, as observed by echocardiography (Supplementary Video S1). Obtaining accurate transvalvular gradients on echocardiography proved unreliable, and this parameter was obtained with direct invasive measurement prior to euthanasia. As can be observed in Figure 5, the gradients remained unchanged from implantation to explantation except for the Glycar valves, where the gradient increased numerically from a mean of 12 to 37 mmHg, although this was not statistically significant.

Macroscopic evaluation of the valves indicated that the stents remained structurally unchanged from implantation. Glycar leaflets were rigid with nodules of calcifications and thickened by pannus overgrowth. DE leaflets were soft, pliable, and non-calcified. DF leaflets were pliable, non-calcified, and of the same thickness as at implantation. An X-ray of the explanted valves showed calcification of the Glycar leaflets. The DE and DF leaflets were generally free from calcification, although there were a few spots next to the stent frame that could represent calcification due to suture trauma (Figure 4).

**Figure 6:**
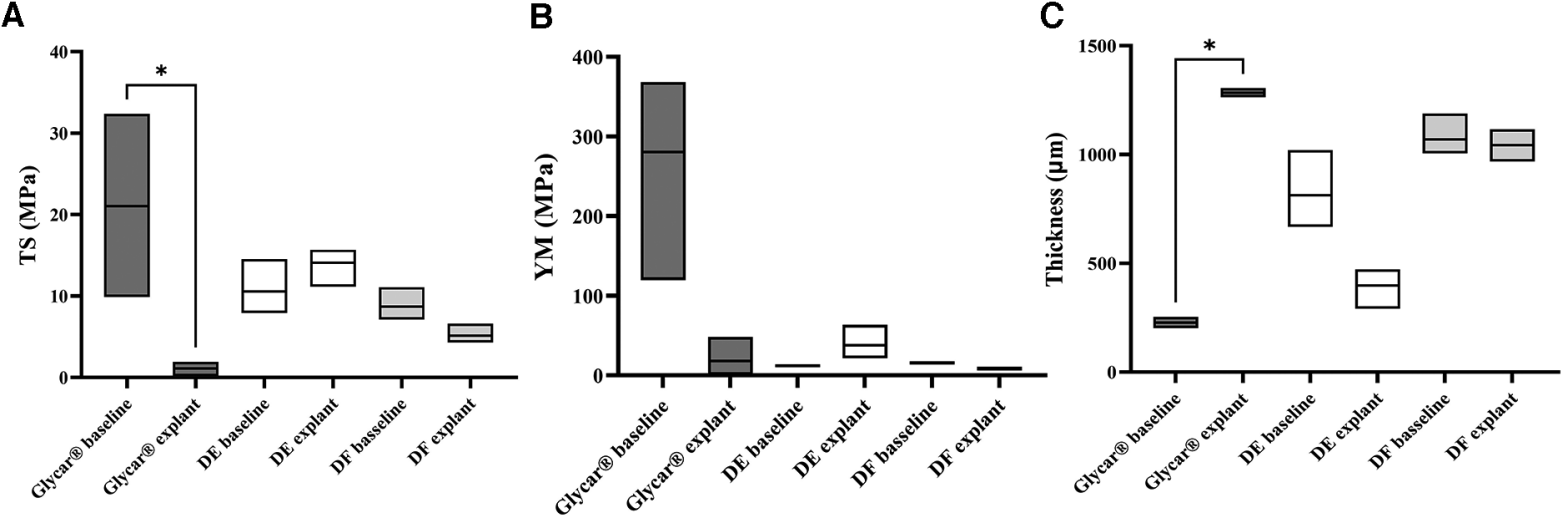
Physical properties of the tissue expressed as medians and ranges. (**A**) TS. Note the significant (*p = 0.033) difference between the Glycar baseline and at explantation. This is due to a single Glycar sample that was severely calcified and fractured with minimal traction. The other tissues all had non-significant differences from baseline to explantation. (**B**) YM analysis with no differences between baseline and explantation. (**C**) Tissue thickness, with only the Glycar tissue showing a significant (*p = 0.0249) change from baseline to explantation. As detailed in the Materials and Methods section, the thickness was measured (physically with calipers) in four places on the leaflet; therefore, a more representative of actual thickness than the single histology slices is shown in Figures 7, 9, and 11. DE denotes decellularized bovine pericardium; DF denotes decellularized, low-dose glutaraldehyde-fixed, and detoxified bovine pericardium; Glycar is the commercially available Glycar bovine pericardium fixed with conventional high-dose GA.

There was no difference in thickness, tensile strength, and Young's modulus from baseline to explantation in either of the two decellularized tissues (DE and DF). The Glycar tissue presented a significant reduction in tensile strength. This was caused by one of the tissues of the Glycar valve that fractured at low traction forces (Figure 6).

**Figure 7:**
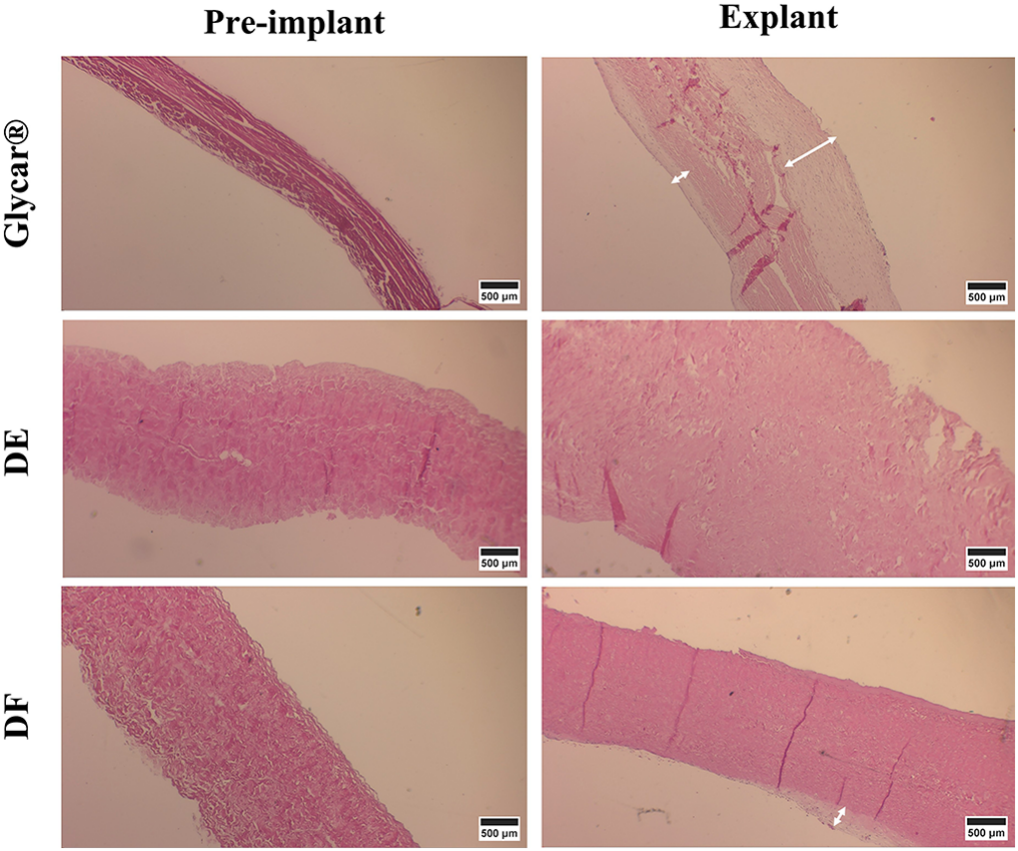
Results of H&E staining of the pericardial tissue at baseline (left) and explantation (right). At the explant, the Glycar tissue has a thick layer of pannus (white arrows), the DF has minimal pannus, and the DE tissue has no pannus. DE denotes decellularized bovine pericardium; DF denotes decellularized, low-dose glutaraldehyde-fixed, and detoxified bovine pericardium; Glycar is the commercially available Glycar bovine pericardium fixed with conventional high-dose GA.

**Figure 8:**
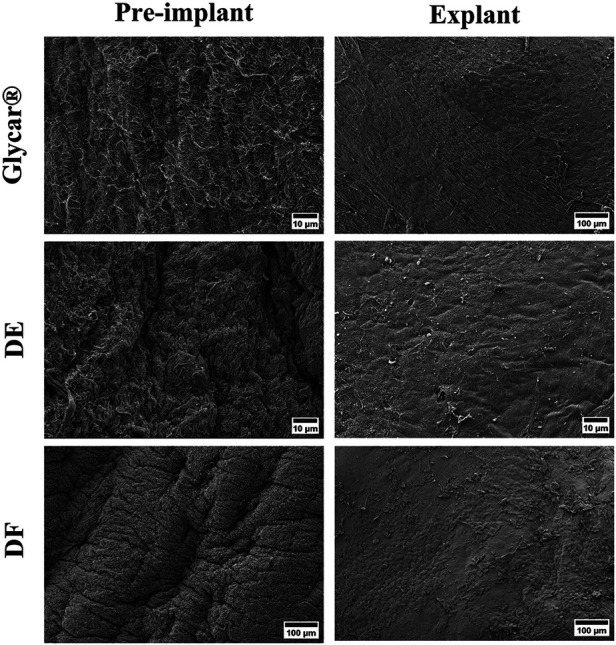
Results of scanning electron microscopy of the pericardial tissue at baseline (left) and explantation (right). All three tissue types were well covered with endothelium at the explant. DE denotes decellularized bovine pericardium; DF denotes decellularized, low-dose glutaraldehyde-fixed, and detoxified bovine pericardium; Glycar is the commercially available Glycar bovine pericardium fixed with conventional high-dose GA.

H&E staining demonstrated pronounced pannus overgrowth of the Glycar leaflet tissue. Of seven sections evaluated, six (86%) had confluent pannus and one (14%) had non-confluent pannus. No pannus was observed in the DE group and the DF group; of four sections, three (75%) had non-confluent pannus and one (25%) had none. In DE explants, no inflammatory process could be identified. There were no inflammatory infiltrates in DF explants, apart from the pannus mentioned previously. In fact, apart from the endothelium and the non-confluent pannus in the DF tissue, these explants were acellular. All tissues had good endothelial coverage, although the endothelium covered the pannus in the Glycar leaflets (Figures 7, 8) and in some portions of the DF leaflets. Scanning electron microscopy confirmed good reendothelialization of all three tissue types at the explant (Figure 8).

**Figure 9:**
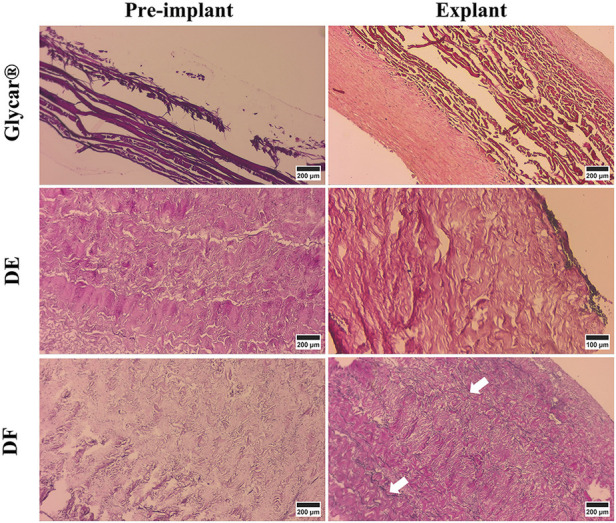
Results of EVG (highlighting elastin) of pericardial tissue at baseline (left) and explantation (right). All tissues had elastin at baseline, but at the explant, DE and Glycar had none and DF had minimal elastin (white arrows). DE denotes decellularized bovine pericardium; DF denotes decellularized, low-dose glutaraldehyde-fixed, and detoxified bovine pericardium; Glycar is the commercially available Glycar bovine pericardium fixed with conventional high-dose GA.

**Figure 10:**
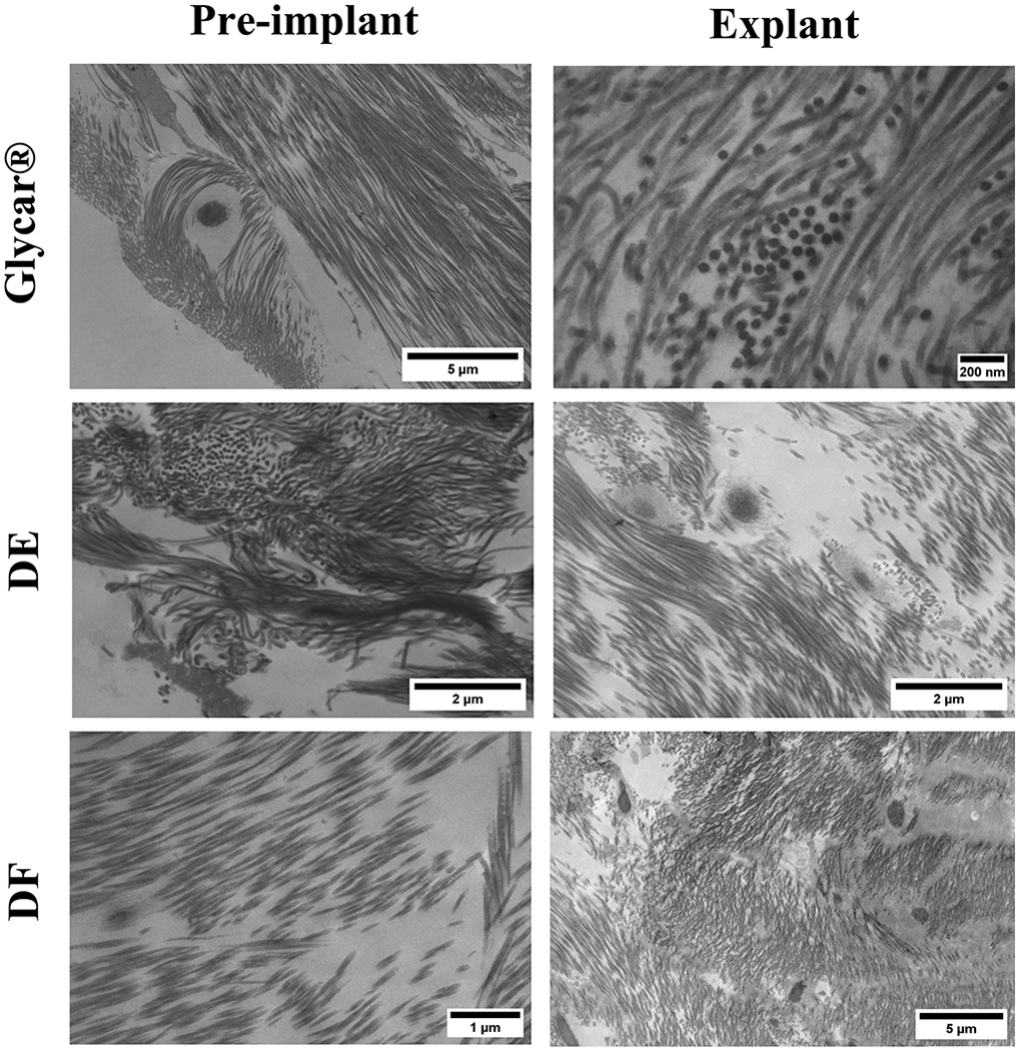
Results of transmission electron microscopy of pericardial tissue at baseline (left) and explantation (right). Note the preservation of collagen structure in all tissues at explantation. DE denotes decellularized bovine pericardium; DF denotes decellularized, low-dose glutaraldehyde-fixed, and detoxified bovine pericardium; Glycar is the commercially available Glycar bovine pericardium fixed with conventional high-dose GA.

Evaluation of the elastin content of the tissues with Verhoeff–Van Gieson staining revealed a dense presence in the Glycar tissue pre-implant while limited presence in the DE and DF tissues. All tissues showed little or no elastin upon explantation (Figure 9). Transmission electron microscopy demonstrated a well-preserved collagen framework in all tissues, closely resembling preimplantation (Figure 10).

**Figure 11:**
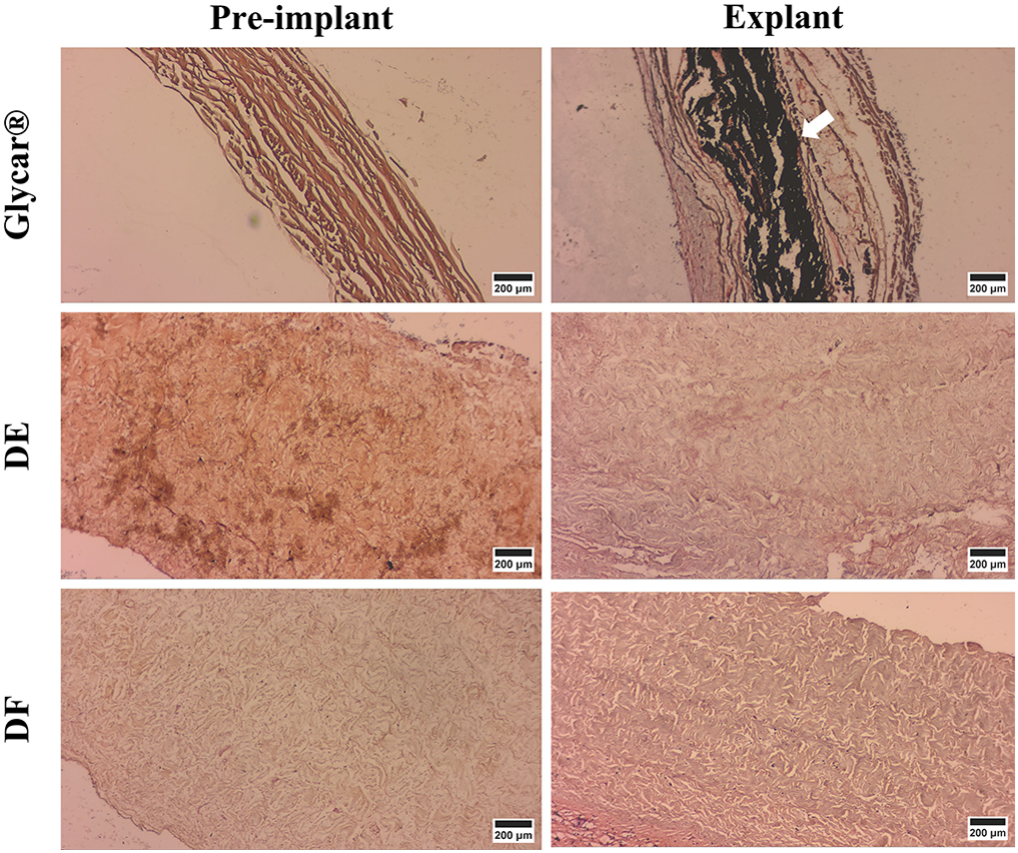
Representative von Kossa-stained samples (indicating calcification in black) of pericardial tissue at baseline (left) and explantation (right). Note the dense calcification in the Glycar tissue (white arrow) at the explant and none in the decellularized tissues. DE denotes decellularized bovine pericardium; DF denotes decellularized, low-dose glutaraldehyde-fixed, and detoxified bovine pericardium; Glycar is the commercially available Glycar bovine pericardium fixed with conventional high-dose GA.

The Von Kossa staining results demonstrated marked calcification in the Glycar tissue and none in the DE and DF groups (Figure 11).

## Discussion

We aimed to develop bioprosthetic THV leaflets with improved durability and potential use in younger patients. Both the decellularized tissues (DE and DF) showed promising results when implanted for 180 days in juvenile sheep. The structure of the DE tissue remained intact, elicited no detectable inflammatory response, and showed no calcification. When a very low dose of GA was added, the DF tissue developed small non-confluent areas of pannus. No calcification was seen during histological examination of the DF valve, and the small spots of calcification seen on an X-ray were near the stent frame and interpreted as a consequence of suture trauma. Elastin, as expected from previous work by others (58), was largely absent from all tissues at the explant.

During preparation of bovine pericardial leaflets, we focused on three aspects that are deemed important in the search for more durable tissues for bioprosthetic THVs in younger patients, namely, lower-dose GA exposure, detoxification, and decellularization.

GA has been used in the fixation of pericardium for bioprosthetic heart valves since the 1970s (59) and has remained a crucial step in the preparation of tissues for heart valves. Currently, bovine pericardium is fixed with GA to provide greater mechanical stability, improve tissue handling, and reduce antigenicity, with a standard concentration of 0.625% used (60). Despite the beneficial effects of GA fixation, it is also implicated in various detrimental effects on the durability of the tissue, including reduced endothelial coverage (61), increased calcium influx into cells (62), inflammatory cell infiltration into tissues (61), and pannus formation (38). We hypothesized that a reduced concentration and the use of monomeric GA will at least partially mitigate some of these effects while providing adequate cross-linking. During the development of our proprietary fixation technique, it was found that the degree of cross-linking reduced with reducing doses of GA. Cross-linking was calculated as the ratio of the bound amino groups in the cross-linked (fixed) samples to the free amino groups from unfixed tissues and was determined using the ninhydrin assay (63). However, when the tissue was exposed to H_2_O_2_, the cross-linking with a very low dose of monomeric GA (0.05%) was similar to that with standard 0.625% GA fixation. Further support that our cross-linking process was adequate can be deducted from the fact that the tensile strength of the DF tissue remained unchanged from baseline to explantation. We have previously shown that Glycar tissue developed pannus in the ovine aorta and ovine pulmonary artery, but in these vascular locations, the Glycar tissue did not calcify excessively (38). Although Glycar tissue is suitable for use in vascular and valvular repair and congenital repair, the current study suggests that it is not suitable for constructing leaflets for a THV. The major differences that the tissues are exposed to in the two locations include the initial potential injury caused by the crimping and balloon expansion of the THV and the repetitive motion and associated mechanical strain experienced by the valvular leaflets, forces that do not occur in vascular grafts. Furthermore, the pannus observed in the Glycar tissue may also have reduced the flexibility of the leaflets, thereby placing more mechanical stress on them, leading to calcification. The lack of calcification in the DF and DE tissues, where minimal or no pannus developed, is in keeping with this interpretation. Although the exact mechanism of calcification in the Glycar tissue remains speculative, this study supports the notion that reducing the exposure to GA limits calcification, particularly when the tissue is exposed to high mechanical stresses such as crimping and expansion of a THV and functioning as a valve leaflet *in vivo*.

Inflammation is widely viewed as an important contributor to bioprosthetic heart valve degeneration (64–67). Pannus tissue contains a variety of chronic inflammatory cells (lymphocytes, plasma cells, macrophages, and foreign body giant cells) and represents a host reaction against a foreign material (67). We did not see an acute inflammatory response to any of the tissues, but the extent of pannus formation was significant in Glycar (with high-dose GA exposure), very little in DF tissue (with a very low dose of GA exposure), and absent in DE (with no GA exposure). Since there were other differences in tissue preparation, we cannot conclude that GA exposure was the sole explanation for this finding. The results would however suggest that any use of GA might have to be avoided, or alternatively, the fixation technique requires further refinement to avoid pannus formation, which could alter hemodynamics and ultimately lead to valve degeneration and calcification. Another factor that has been shown to contribute to the immunogenic response to implanted bioprosthetic leaflets is cellular remnants of bacteria on the tissue (65). However, we did not test for this prior to implantation.

Decellularization in bioprosthetic tissues has been utilized and evaluated with the potential benefit of removal of immunogenic components and reducing stimulus for calcium influx into the tissue (68–72). Decellularized allografts (without GA fixation) have been evaluated in humans (73) with good midterm results. In this cohort of younger patients, one patient required a reoperation after 18 months for another indication and a small portion of the allograft tissue was removed at the time. Histology revealed well-preserved collagen fibers in the media and intimal hyperplasia of moderate intensity. There were a limited number of fibroblasts in the media and minimal inflammatory cells. The concept of utilizing decellularization without fixation is therefore not new, but its utility for xenografts is less well established, with some discouraging results described (74). Our two decellularized tissues showed very encouraging results, but because of the proprietary preparation process, these results should not be generalized to other decellularization protocols or other implantation techniques and animal models. Decellularization can be obtained through various methods (physical, enzymatic, or chemical) but needs to be tailored for each tissue type depending on cellularity, density, lipid content, and thickness (75). Each step of the process has different effects on the outcome (36), and related techniques may yield different results depending on small variations in the technique. Our decellularization methodology has been developed over various iterations, and the results reported here are from tissues with encouraging performance in the subcutaneous rat model and aortic and pulmonary artery ovine implants (38). However, the durability of the unfixed DE tissue after 6 months in the high mechanical stress environment of a THV in sheep is somewhat surprising. No host fibroblast ingrowth was seen in this tissue, which implies that the collagen structure from the implant was still sufficiently stable, as seen from the TEM images, and remained functional, as demonstrated by the tensile strength and Young's modulus evaluation. This is contrary to the current view that regenerative tissue engineering supplies a scaffold for the host tissue to infiltrate, produce new structural elements, and eventually take over the functioning of the original xenograft (76). Although recellularization is an accepted endpoint in most tissue-engineered scaffolds, no one has been able to stimulate pericardial tissue to regenerate into the complex and specialized three-layered structure of native aortic valve leaflets. Although researchers have been able to populate scaffolds with the appropriate cells (*in vivo* and *in vitro*), they have been unable to stimulate them to produce a new extracellular matrix with mature composition, distribution, and conformation (76). Our decellularized tissue, conversely, was essentially inert despite 6 months of exposure to an accelerated calcification model (juvenile sheep). This finding is unique and challenges conventional dogma. Further research is required to fully understand this finding and its potential impact on future transcatheter valves. Each tissue was tested on three animals only, which makes the data preliminary and exploratory at best. However, because of the encouraging results, they justify expanding it to a larger cohort. The results were obtained in the lower-pressure environment and must be validated with aortic implants. The encouraging results in the RVOT, however, raise the question of whether the tissue is suitable for pulmonary THVs in younger patients. To evaluate long-term mechanical integrity of the tissue, our valves are currently being fatigue-tested to 200 million cycles per ISO 5840 standards. Longer-term implants will be required to prove durability, especially for the unfixed DE tissue. Finally, the response of the tissue to anti-Gal antibodies was not tested, and primate implants or *in vitro* exposure to human tissue must be performed.

Another feature of our work that is relatively unique is that valves were implanted in a synthetic tube and therefore (although exposed to host blood) not in direct contact with host tissue. This may be part of the explanation for the lack of host cell infiltration in the decellularized tissues, although it did not protect the Glycar tissue from pannus formation nor the DF group from limited non-confluent pannus formation. Applying this concept of limiting host tissue contact to aortic valves will be difficult because of the coronary ostia, but it may have utility in pulmonary valve implants where the recipients also tend to be younger.

## Conclusions

In the juvenile sheep THV model, Glycar tissue (with high-dose GA fixation) developed significant pannus, calcification, and hemodynamic deterioration. Using a very low dose of monomeric GA to fix decellularized bovine pericardium yielded less pannus formation, less calcification, and better hemodynamic functioning. We postulate that the limited pannus formation in the DF group results from GA, as no cellular response or pannus formation was demonstrated when GA was omitted in the similarly decellularized DE tissue. Bovine pericardium decellularized with our proprietary method resulted in essentially inert tissue, which is a unique finding. These results justify further development and evaluation of the two decellularized tissue types in THVs for use in younger patients.

## Data Availability

The original contributions presented in the study are included in the article/**Supplementary Material**, further inquiries can be directed to the corresponding author.

## Supplementary material

Supplementary Video S1 Representative echocardiogram [in a view comparable to a parasternal short axis view] showing color Doppler over the right ventricular outflow and pulmonary artery with no regurgitation of the valve in situ.
